# Intraperitoneal Inoculation: An Atypical Route of *Trichinella spiralis* Infection

**Published:** 2017

**Authors:** Peng JIANG, Zi Fang ZHANG, Zhong Quan WANG, Ruo Dan LIU, Xi ZHANG, Ge Ge SUN, Xin QI, Li WANG, Jing CUI

**Affiliations:** Dept. of Parasitology, Medical College, Zhengzhou University, Zhengzhou, P. R. China

**Keywords:** *Trichinella spiralis*, Infection route, Intraperitoneal injection, Mechanical force

## Abstract

**Background:**

The intraperitoneal injection is a common method for establishing the experimental animal model infected with parasites. The aim of this study was to investigate if the intraperitoneal injection was another route of *Trichinella spiralis* infection.

**Methods:**

From June to July 2015, twenty BALB/c mice were intraperitoneally injected with 300 *T. spiralis* muscle larvae in Department of Parasitology, Medical College, Zhengzhou University, China. The larvae per gr (LPG) muscle from the infected mice and the reproductive capacity index (RCI) of *T. spiralis* were calculated

**Results:**

Sixty percent (12/20) mice injected were successfully infected at 35 day post injection (dpi), but the muscle larval burden (381.53 larvae per gr) and reproductive capacity index (32.33) in infected mice was lower.

**Conclusion:**

A mechanical force indicated as a possible mechanism in successful larval invasion of almost all kind of host tissues. However, the exact migratory route of larvae from peritoneal cavity into small intestine is not clear.

## Introduction

*Trichinella spiralis* is a tissue-dwelling parasitic nematode infecting more than 150 kinds of animals and is the main aetiological agent of trichinellosis. Once ingested, *T. spiralis* muscle larvae are released from the capsules under the action of gastric juice, then penetrate into the intestinal epithelial cells (IECs), establishing an intramulti-cellular niche which composed of numerous IECs, where they developed into adult worms, mate and reproduce, yielding the next generation of larvae (1, 2). The life cycle of *T. spiralis* completed when newborn larvae developed into the encapsulated larvae in skeletal muscles (3).

*T. spiralis* infection is mainly caused by ingesting raw or undercooked meat from infected animals. However, the transplacental transmission of *Trichinella* larvae has been recorded in few animals such as pigs, rabbits, rats, mice, and humans (4–6). In addition, the experimental *T. spiralis* infection was established by intravenous injection of the newborn larvae (7). The intraperitoneal injection is the common method for establishing the experimental animal model infected with parasites, such as *Toxoplasma gondii* (8), *Leishmania donovani* (9), *Paragonimus* (10), *Echinococcus granulosus* (11), etc. Nevertheless, the intraperitoneal injection with *T. spiralis* was not reported, yet.

The aim of the present study was to observe if the mice were infected by intraperitoneal injection with *T. spiralis* muscle larvae and investigate the possible migratory route in infected mice.

## Materials and Methods

### Ethics statement

The Life Science Ethics Committee of Zhengzhou University (Permission No. 2012-0009) approved all procedures of animal experiment.

### Parasite and experimental animals

In March 1987, *T. spiralis* (ISS534) were obtained from domestic pigs in Nanyang, Henan Province of China in Department of Parasitology, Henan Medical College, China. The isolate was maintained by specific pathogen free (SPF) BALB/c mice (SCXK 2010-0002), it is correct purchased from the Experimental Animal Center of Henan Province, and was housed under specific pathogen free conditions including a filtered atmosphere.

### Intraperitoneal injection

*T. spiralis* muscle larvae were collected from infected mice at 35 d post infection (dpi) by artificial digestion method as previously described (12, 13). Twenty mice were used and each was intraperitoneally inoculated with 300 muscles larvae. The infected mice were sacrificed at 35 d post inoculation (dpi) by exposure to ether and cervical dislocation, and their carcasses were inspected by compression method and digestion method. The larvae per gr (LPG) muscle from the infected mice and the reproductive capacity index (RCI) of *T. spiralis* were calculated and shown as the mean ± standard deviation (SD) (14, 15).

### Examination of T. spiralis larvae in peritoneal lavage

Another 40 mice were randomly divided into 4 groups of 10 mice, and each mouse was intraperitoneally injected with 300 muscles larvae. Before aspiration, all the inoculated mice were intraperitoneally injected with 5 ml saline, and then put the mouse upside down for 5 times, the peritoneal lavage fluid was aspirated at 5, 10, 15 and 30 min post injection (mpi) for detecting the larvae, respectively.

### Compression method and immunohistochemistry

Additional 30 mice were randomly divided into 3 groups of 10 mice, and each mouse was intraperitoneally injected with 300 muscles larvae, and sacrificed at 15, 30, and 45 min, and then hourly during 1–12 h post injection. The mesentery, small intestine, liver, spleen, diaphragm, lung, and abdominal muscles of inoculated mice collected, washed, and examined for *T. spiralis* larvae. By compression method the above mouse organs were fixed with formaldehyde and embedded in paraffin, then the 5 μm sections of tissues were blocked with normal goat serum (1: 10) for 30 min, and incubated with sera of mice infected with *T. spiralis* (1: 200 dilution) at 37 °C for 1 h. The sections washed with PBS and subsequently incubated with a 1: 500 dilution of HRP-labeled goat anti-mouse IgG (Sigma, USA) at 37 °C for 40 min. After being washed with PBS, the sections were reacted with 3, 3′-diaminobenzidine (DAB) for 5 min, and observed under light microscope (16).

### Larval invasion in agarose gel

**Fig. 1:**
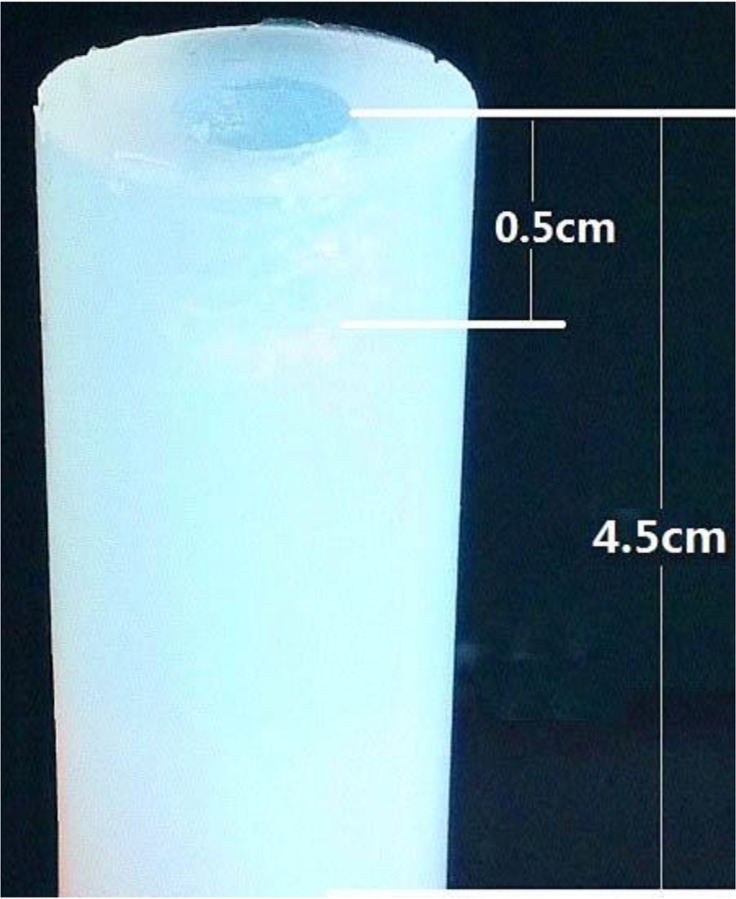
Agarose gel cylinder for testing mechanical force of *T. spiralis* larval invasion

To test the larval mechanical force during invasion, different concentrations of agarose gels (0.5%, 1.0%, 1.5% and 2.0%) were prepared. The melted agarose poured into a cylindrical cast (4.5 cm thick, 1 cm in diameter). A small hole (0.5 cm in deep and diameter) dug in the central top of the gel column (Fig. 1). About 1000 ML were added into the hole of gel, the numbers of ML that passed through the gel after 5 h were counted under an Olympus microscope. The gel invaded by ML was cut to the sections of 1 mm thickness and larvae arrested in the gel was examined and counted.

### Statistical analyses

All statistical analyses of data were done with PASW ver. 18.0 (IBM SPSS Statistics, Chicago, IL, USA). The trend of larval recovery rates in mouse peritoneal lavage at different time post injection and the larval penetration rates among different concentration of gels were tested by Chi-square test, respectively. The Pearson correlation was used to analyze the correlation between the larval penetration rates in gel and the gel concentrations. The level of significance used was 5% (*P*<0.05).

## Results

### The T. spiralis infection rates of intraperitoneally injected mice

The infection rate of mice intraperitoneally injected with 300 *T. spiralis* ML was 60% (12/20), respectively. The muscle larval burden in successfully infected mice was 381.53±511.67, ranging from 3.71 to 1062.31 larvae per gram. The RCI of *T. spiralis* in infected mice was 32.33±45.23, varied from 0.73 to 127.27.

### Examination of the larvae in peritoneal lavage of injected mice

The detection rate of *T. spiralis* larvae in peritoneal lavage of injected mice at 5, 10, 15, and 30 mpi was 100% (10/10), 100% (10/10), 80% (8/10) and 0% (0/10), respectively. The total larval recovery from peritoneal lavage was 3.63% (109/3000), 1.77% (53/3000) and 0.57% (17/3000) and 0% (0/3000) at 5, 10, 15, and 30 mpi, respectively. The larval recovery rates had an obvious descending trend along with the prolongation of time post injection (*χ*^2^=172.895, *P*<0.01).

### Examination of the larvae in tissues by compression and immunohistochemistry

**Fig. 2:**
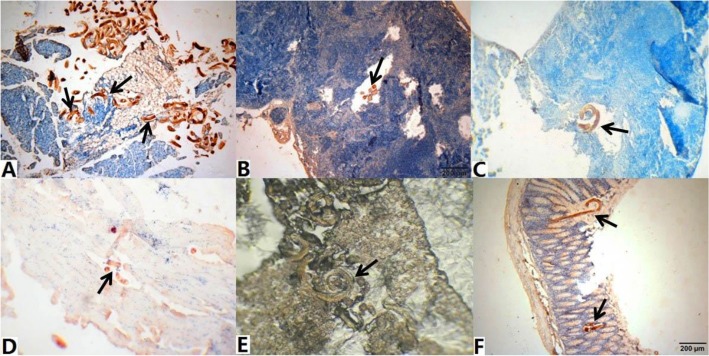
*T. spiralis* larvae in different organs/tissues of intraperitoneally injected mice (Immunohistochemistry staining, 40×) A. mesentery; B. liver; C. spleen; D. diaphragm; E. lung; F. small intestine -Arrows showed the larvae in tissue sections.

The results of tissue compression and immunohistochemistry in injected mice showed that *T. spiralis* larvae were found in the mesentery, liver, spleen, abdominal muscle, diaphragm and lung of infected mice at 15 mpi (Table 1, Fig. 2A–2E). On immunohistochemistry staining, the larvae were found in small intestinal epithelium at 12 h after injection (Fig. 2F), further demonstrated that the mice were infected with *T. spiralis* by intraperitoneal injection.

**Table 1:** The number of *T. spiralis* larvae in different organs/tissues of intraperitoneally injected mice by compression method

**Time post injection (min)**	**Number of larvae in organs/tissues**						
	**mesentery**	**liver**	**spleen**	**pancreas**	**abdominal muscle**	**diaphragm**	**lungs**
15	37	13	5	4	20	2	1
30	52	26	13	2	16	8	6
45	28	4	2	8	63	0	1
total	117	43	20	14	99	10	8

### Larval invasion in agarose gel

The rate of larvae penetrated and passed through 0.5%, 1.0%, 1.5% and 2.0% gels was 64.9, 53.1, 37.3 and 16.7%, respectively (*χ*^2^ =532.762, *P*<0.01). Table 2, suggested that the ML could pass through agarose gel column with 4 cm thick, and the larval passing rate had a descending trends with increasing gel concentration (*χ*^2^=524.719, *P*<0.01) and obvious negative correlation with the gel concentrations (*r*=−0.993, *P*<0.01). The rate of larvae invaded into and arrested in 0.5%, 1.0%, 1.5% and 2.0% gels was 33.2, 34.1, 42.0 and 46.1%, respectively (*χ*^2^ =49.236, *P*<0.01). The larval invasion rate in the gel had descending trends with increasing gel concentration (*χ*^2^=45.693, *P*<0.01) and obvious negative correlation with the gel concentrations (*r*=−0.963, *P*<0.05).

**Table 2:** The number of *T. spiralis* larvae passed through and invaded the agarose gel within 5 h

**Agarose gel concentration (%)**	**No. of larvae added in central top hole of gel column**	**No. of larvae passed through gel (%)**	**No. of larvae invaded gel (%)**	**No. of residual larvae in central top hole of gel column (%)**
0.5	1000	649 (64.9)	332 (33.2)	19 (1.9)
1.0	1000	531 (53.1)	341 (34.1)	128 (12.8)
1.5	1000	373 (37.3)	420 (42.0)	207 (20.7)
2.0	1000	167 (16.7)	461 (46.1)	372 (37.2)

## Discussion

After the infected meat containing the muscle larvae is ingested, the key step for *T. spiralis* infection is the larval invasion of host intestinal epithelial cells (17, 18). In this study, the intraperitoneal inoculation with muscle larvae as an atypical and abnormal route of *T. spiralis* infection was confirmed. Since the intraperitoneal inoculation is not a natural route of infection, the infection rate of intraperitoneally inoculated mice was only 60%. Besides, the muscle larval burden and reproductive capacity index in the intraperitoneally infected mice was lower than those in orally infected mice at 35 dpi (14, 19, 20).

As shown in Fig. 1, the intraperitoneally injected larvae invaded the mesentery, liver, spleen, abdominal muscle, diaphragm, lung, and finally entered small intestines of inoculated mice. *T. spiralis* muscle larvae injected subcutaneously could invade the myofibers 14 d after injection and developed into the nurse cell-larva complex (21). Additionally, most of the larvae penetrated into or passed through the agarose gels columns with 4 cm thick in this study. The migratory larvae of *T. spiralis* could go through the placental barrier and infected the conceptus in rats and mice (4, 22).

The mechanical force seemed to be the important factor for larval invasion of host different tissues. *T. spiralis* larvae observed in different tissues of inoculated mice by compression and/or immunostaining only 15 mpi, and then found in small intestinal epithelium at 12 h post injection. The larval successful invasion of different tissues indicated that the larvae have an obvious invasive ability. Because intraperitoneal inoculation is not a normal route of infection, after being injected into the peritoneal cavity, the larvae were exposed to a new and unfamiliar milieu in which the larvae could not migrate and develop in accordance with normal life cycle of *Trichinella*.

The low infection rate and RCI in infected mice suggested that the inoculated larvae had no definite route of migration, the larvae might accidently invade small intestine from the peritoneal cavity by larval mechanical penetration. However, the exact migratory route of the inoculated larvae from peritoneal cavity into small intestine is not clear. The possibility larvae injected into the lumen of small intestine should be excluded due to precautions that took place during the manipulation of animals. The larval size (about 1.0 mm×0.03 mm) did not permit them to migrate via blood circulatory system. Because the larvae had a strong penetration force and could pass through the gel column, we assumed firstly that the larvae penetrated through directly intestinal wall. However, no obvious focal hemorrhage and inflammation was seen in small intestine. Moreover, all the larvae in intestine seemed to invade the mucosa from intestinal lumen instead of serosal and muscular layer as shown in Fig. 1F. Considering that the larvae were found in diaphragm and lung of inoculated mice, the inoculated larvae invaded diaphragm, lung, and then might enter the alveoli. The larvae are likely to move up the respiratory tree and trachea to the epiglottis to be swallowed, and finally enter into small intestine. The larvae in liver might enter into small intestine via bile duct.

## Conclusion

Although the mice can be infected by intraperitoneal inoculation with *T. spiralis* muscle larvae, the infection rate, muscle larval burden and reproductive capacity index in the intraperitoneally infected mice was lower than those in orally infected mice. The intraperitoneal inoculation is an atypical and abnormal route of *T. spiralis* infection, but the exact migratory route of larvae from peritoneal cavity into small intestine is not clear.
